# The formalin test does not probe inflammatory pain but excitotoxicity in rodent skin

**DOI:** 10.14814/phy2.15194

**Published:** 2022-03-27

**Authors:** Tal Hoffmann, Florian Klemm, Tatjana I Kichko, Susanne K Sauer, Katrin Kistner, Bernhard Riedl, Patrick Raboisson, Lei Luo, Alexandru Babes, Laurence Kocher, Giancarlo Carli, Michael J. M. Fischer, Peter W. Reeh

**Affiliations:** ^1^ Institute of Physiology and Pathophysiology University of Erlangen‐Nürnberg Erlangen Germany; ^2^ Institute of Physiology and Pathophysiology University of Heidelberg Heidelberg Germany; ^3^ AstraZeneca, CNS and Pain Innovative Medicines Unit Södertälje Sweden; ^4^ Department of Anatomy, Physiology and Biophysics University of Bucharest Bucharest Romania; ^5^ Laboratoire de Physiologie Centre Hospitalier Lyon Sud Faculté de Médecine Université de Lyon France; ^6^ Department of Physiology Università degli Studi di Siena Siena Italy; ^7^ Center of Physiology and Pharmacology Medical University of Vienna Vienna Austria

**Keywords:** analgesia, formaldehyde, nociception, toxicokinetics, TRPA1

## Abstract

The most widely used formalin test to screen antinociceptive drug candidates is still apostrophized as targeting inflammatory pain, in spite of strong opposing evidence published. In our rat skin‐nerve preparation *ex vivo*, recording from all classes of sensory single‐fibers (n = 32), 30 units were transiently excited by formaldehyde concentrations 1–100 mM applied to receptive fields (RFs) for 3 min, C and Aδ‐fibers being more sensitive (1–30 mM) than Aβ−fibers. From 30 mM on, ~1% of the concentration usually injected *in vivo*, all RFs were defunctionalized and conduction in an isolated sciatic nerve preparation was irreversibly blocked. Thus, formaldehyde, generated a state of ‘anesthesia dolorosa’ in the RFs in so far as after a quiescent interphase all fibers with unmyelinated terminals developed a second phase of vigorous discharge activity which correlated well in time course and magnitude with published pain‐related behaviors. Sural nerve filament recordings *in vivo* confirmed that higher formalin concentrations (> 42 mM) have to be injected to the skin to induce this second phase of discharge. Patch‐clamp and calcium‐imaging confirmed TRPA1 as the primary transducer of formaldehyde (10 mM) effects on mouse sensory neurons. However, stimulated CGRP release from isolated skin of TRPA1^+/+^ and TRPA1^–/–^ mice showed a convergence of the saturating concentration‐response curves at 100 mM formaldehyde, which did not occur with nerve and trachea preparations. Finally, skin‐nerve recordings from C and Aδ‐fibers of TRPA1^–/–^ mice revealed a massive reduction in formaldehyde (30 mM)‐evoked discharge. However, the remaining activity was still biphasic, thus confirming additional unspecific excitotoxic actions of the fixative that diffuses along still excitable axons as previously published. The multiplicity of formaldehyde's actions requires extensive discussion and literature review, leading to a fundamental reevaluation of the formalin test.

## Graphical abstract

The widely used formalin test to screen analgesic drug candidates in rodents does not probe inflammatory pain, as it destroys the sensory nerve endings at the injected skin site and causes sustained pain just by the rapid diffusion of toxic formaldehyde along still excitable nerve fibers.

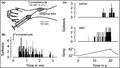

## 1 INTRODUCTION

The introduction of the formalin test in 1977 to screen drugs for analgesic activity and explore their action mechanism has led to a steep wave of 19,500 pertinent papers listed in Google Scholar (Dubuisson & Dennis, 1977). A major review article published a table with nearly two hundred drugs mitigating the distinctly biphasic time course of formalin‐induced pain‐related behavior in rodents (Porro & Cavazzuti, 1993). A considerable proportion of these drugs never came into question as analgesic, for example verapamil, amphetamine, neomycin, propranolol, atropine, L‐tryptophan; they obviously affected the formalin test by other than antinociceptive effects. However, this lack of predictive power did not essentially curb the confidence of researchers who were particularly inspired by the prolonged second phase of pain behavior. The latter seemed to require an explanation independent of the short first phase that was attributed to a putative injury discharge of nociceptive nerves. The hypothesis, that spinal dorsal horn neurons developed a life of their own after being sensitized by the injury discharge, was undermined when it was shown that a short‐acting local anesthetic co‐injected with formalin and precluding the first phase could not prevent the second phase to build up (Dallel et al., 1995). In addition, the second phase could be terminated at any time by sciatic nerve block (Pitcher & Henry, 2002). The other hypothesis, prevailing to date, regards the second phase as a result of inflammatory sensitization of skin nerve endings, although an increased sensitivity or excitability in the formalin‐infiltrated skin could never actually be demonstrated during the fifty and more minutes of animals’ pain‐related agitation (Tjølsen et al., 1992). All theories pass over the fact that only rats, mice, and guinea pigs show biphasic pain in the formalin test, whereas larger mammals such as cats, rabbits, dogs, and monkeys present with a monophasic decrescendo behavior lasting up to sixty and more minutes (Aloisi et al., 1993; Alreja et al., 1984; Dubuisson & Dennis, 1977; Leite‐Panissi et al., 2001).

This conspicuous species difference cannot easily be explained by our recent work on the formalin test. However, it provided convergent evidence that it is the high diffusion rate of the micromolecule formaldehyde (~ 30 g/mol) which accounts for the long duration of its expanding excitotoxic, primarily TRPA1‐mediated, action along excitable nerve fibers, leaving “scorched earth” behind and coming to an end by dilution and clearance, dose‐dependently after 40–60 min (Fischer et al., 2014, 2015; Macpherson et al., 2007; McNamara et al., 2007). After the initial transient depolarisation and excitation, the quiescent interphase in pain‐related behavior around eight minutes after formalin injection seems to result from a distinct hyperpolarizing action, followed by a final inactivation of the neurons related to irreversible block of voltage‐gated sodium channels. Combined computer modeling of the diffusion and electrophysiological processes provided a clearly biphasic time course and magnitude of events which do not require additional parameters of inflammatory or spinal sensitization (Fischer et al., 2014). This confirmed previous single‐fiber recordings from rat cutaneous primary afferents *in vivo* that demonstrated biphasic discharge activity upon formalin injection which, however, could not exclude inflammatory mechanisms to be responsible for the second phase (McCall et al., 1996; Puig & Sorkin, 1996). Of course, inflammation does follow upon the tissue damage caused by the fixative formalin (1–10%), and it results in hyperalgesia and allodynia for at least two weeks (Fischer, 1905). However, the appearance of the classical formalin test is governed by excitotoxicity and toxicokinetics, as we demonstrate in the present paper with a diverse set of experimental data mostly gained *in vitro* from isolated peripheral tissue preparations lacking blood perfusion, platelet activation, and immune cell invasion, thus, the essentials of an inflammatory process.

## 2 MATERIALS AND METHODS

### 2.1 Animals

**FIGURE 1:**
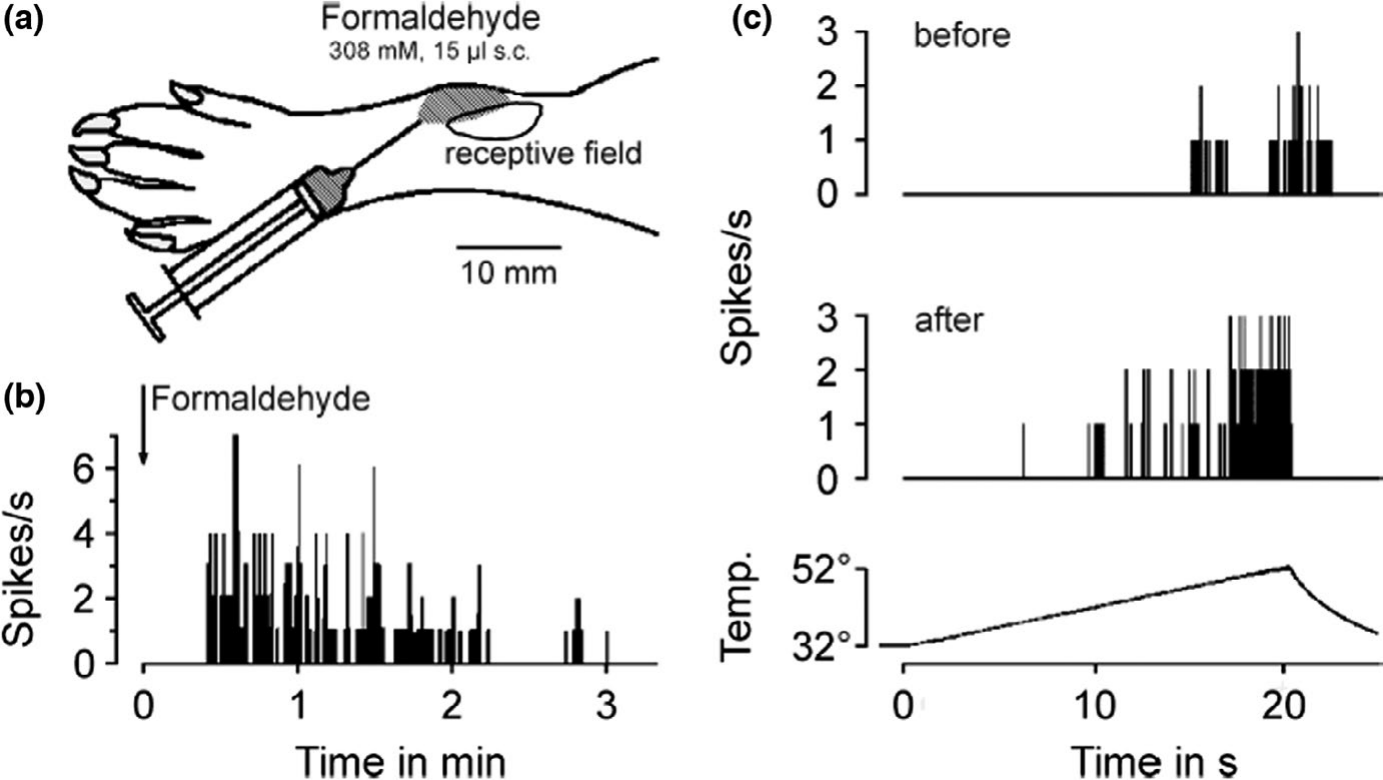
Formaldehyde excites and sensitizes rat cutaneous nociceptors by lateral diffusion *in vivo*. (a) Schematic drawing of the rat hindpaw, showing location and extent of the mechanoreceptive field of a C‐MH fiber (0.85 m/s, threshold 22.6 mN) and the adjacent site of the injection. (b) Formaldehyde was injected to reach the receptive field by diffusion, note the delayed onset of formaldehyde‐induced activity. (c) Activity evoked by a radiant heat ramp to 52°C. Compared to before, the heat response 5 min after injection of formaldehyde was sensitized, although the mechanosensitivity was lost in half of the receptive field (specimen, representative for 3 C‐MH fibers)

According to German law the animal experiments were authorized by the competent district governments (in Karlsruhe, Ansbach, and Würzburg, Germany) after the approval by the pertinent animal ethics commissions. The studies were performed on inbred adult Wistar and Sprague‐Dawley rats and C57BL/6J mice of either sex. Heterozygous TRPV1^+/–^ mice came from John B. Davis (SmithKline Beecham, Harlow, UK), and TRPA1^+/–^ animals were a generous gift from David P. Corey and were back‐crossed on C57BL/6J for at least eight generations (Davis et al., 2000; Kwan et al., 2006). Wildtype littermates and congenic C57BL/6J mice served as control animals. Animal husbandry and breeding were supervised by the local veterinary and animal protection authorities (Heidelberg and Erlangen, Germany). The animals were housed in group cages in a temperature‐controlled environment on a 12 h light‐dark cycle and were supplied with food and water *ad libitum*. Apart from rat pilot experiments and sural nerve recordings *in vivo* (Figures 1 and 4), all other experiments were done *ex vivo*. These animals were euthanized in rising CO_2_ atmosphere. The rat *in vivo* experiments were done in deep anesthesia using thiopental (120 mg/kg i.p., BYK Gulden, Konstanz, Germany) and urethane (11%, 1 ml/kg, Sigma‐Aldrich, Stockholm, Sweden) in Södertälje, Sweden. At the end of these experiments the rats were sacrificed with an overdose of pentobarbital (SERVA, Heidelberg, Germany).

### 2.2 Substances and solutions

Formaldehyde was obtained as a 37% saturated solution of formaldehyde in water (assured concentration 37–37.5 mass%, stabilized by methanol 8–12%, Carl Roth, Karlsruhe, Germany). A stock solution of 1 M formaldehyde was prepared in distilled water and freshly diluted in extracellular solution (in mM: 140 NaCl, 5 KCl, 2 CaCl2, 2 MgCl2, 10 HEPES, 10 glucose, pH 7.4) or **s**ynthetic **i**nterstitial **f**luid (SIF, in mM: 107.8 NaCl, 26.2 NaCO_3_, 9.64 Na‐gluconate, 7.6 sucrose, 5.05 glucose, 3.48 KCl, 1.67 NaH_2_PO_4_, 1.53 CaCl_2_ and 0.69 MgSO_4_, buffered at pH 7.4 by carbogen) (Bretag, 1969). To avoid confusion between % of formalin and % of formaldehyde in water, the concentration of formaldehyde is presented in mM throughout the manuscript. For calcium imaging experiments the TRPA1 agonist acrolein was used (Sigma‐Aldrich, Taufkirchen, Germany).

### 2.3 In vitro single‐fiber electrophysiology

Single‐fiber recordings from mechanosensitive A and C‐fibers of the saphenous nerve in rats and mice were obtained using the isolated skin‐nerve preparation as described previously (Reeh, 1986; Zimmermann et al., 2009). A skin flap of the hairy hindpaw skin in continuity with the innervating saphenous nerve was excised and fixed in an organ bath, corium side up. The chamber containing the skin was continuously superfused with SIF heated to 32°C. The nerve trunk was placed on a mirror in a separate chamber where SIF was overlaid with paraffin oil, desheathed and divided under binocular control until a unit with a single distinguishable receptive field was obtained. Recordings were acquired using the SPIKE/SPIDI software package (Forster & Handwerker, 1990) and a CED Micro1401, running Spike2 software (Cambridge Electronic Design, Cambridge, England). Initially, mechanosensitive receptive fields were mapped using a blunt glass rod. Once a distinct receptive field was identified, a metal microelectrode was placed in the receptive field to measure conduction latency. The calculated conduction velocity was used for fiber classification with a cut‐off criterion of < 1.4 m/s for unmyelinated fibers. A marking technique, in which latency shifts are provoked by simultaneous application of mechanical and electrical stimulation to the receptive field, was then applied (Schmelz et al., 1995) to ensure the identity of the recorded action potentials as originating from only one single‐fiber. For further characterization the mechanical, heat and cold sensitivity of the individual fiber was tested. Each stimulus was followed by a period of several minutes prior to onset of the following stimulus in order to minimize the risk of inter‐modality sensitization or desensitization. First, the mechanical threshold was tested using calibrated von Frey filaments ranging in buckling load from 1–256 mN on a geometric scale. Subsequently, a metal ring of ~9 mm inner diameter was placed above the receptive field. The ring was then evacuated and flushed for cold stimulation with SIF at 4°C for at least 20 s. After evacuation and adaptation to 32°C followed radiant heat stimulation (20 s ramp to 47°C) delivered by a feedback‐controlled halogen lamp with an 8 mm focused beam. Heat threshold was taken as the temperature at which the second spike of the response occurred. The Aδ and C‐fibers were subclassified as mechano‐heat (MH), high‐threshold mechano‐ (HTM), low‐threshold mechano‐ (LTM), mechano‐cold‐ (MC) or mechano‐heat‐cold‐ (MHC) sensitive units, and the low‐threshold mechanosensitive Aα/β fibers (conduction velocity > 12 m/s) were only distinguished as slowly adapting type I (SA I with irregular discharge) and rapidly adapting.

The pilot experiments *in vivo* with formalin 2.5% (corresponding to 308 mM concentration) were done by intradermal injection into or adjacent to receptive fields; this protocol is detailed in the Results section. In the experiments to determine the formaldehyde threshold concentration in the isolated rat skin‐nerve preparation 0.1, 1, 10, 30, 100, and 308 mM formaldehyde was topically applied to the receptive field by filling the metal ring for a period of three minutes. The formaldehyde solution was then removed, the metal ring dislodged, and SIF was allowed to superfuse the receptive field for further three minutes. Formaldehyde‐evoked discharge activity was continuously recorded throughout the application period and thereafter. In case the fiber did not respond, the next higher concentration was applied after the wash‐out period.

### 2.4 Compound action potential electrophysiology in vitro

Compound action potential (CAP) recordings were made from C‐ and A‐fibers of isolated rat sciatic nerves. The sciatic nerve was exposed and excised from its point of emergence out of the lumbar plexus to the trifurcation into tibial, sural and peroneal nerves. The nerve was mounted in a three‐compartment chamber, and the middle compartment was continuously perfused with synthetic interstitial fluid (SIF) or the formaldehyde test solutions at a rate of 5–7 ml/min. Solutions were maintained at pH 7.4 by bubbling with carbogen and temperature was held constant at 32°C. Each end of the nerve was threaded into separate lateral chambers filled with fluorocarbon oil FC‐43 (3 M, USA) and placed on gold wire electrodes. One chamber contained the recording electrodes while the other was used for electrical stimulation. CAP responses supra‐maximal (125%) for the C‐fiber volley were evoked with constant voltage pulses (A395, WPI, Sarasota, USA) of fixed duration (0.1 ms) at a rate of 0.2 Hz, and the responses were monopolarly recorded. After a 20 min control period perfusing SIF, formaldehyde 1, 10 and 30 mM was consecutively perfused for 10 min each and changes in both latency and amplitude of C‐fiber and A‐fiber CAPs were assessed. Data were acquired and analyzed using a CED Micro1401 and Spike2 software (Cambridge Electronic Design, Cambridge, UK). The peak‐to‐peak amplitude of the recorded waveform and the latency to onset of the CAP are displayed over time.

### 2.5 Rat sural neve filament recordings in vivo

First, the saphenous and sciatic nerves were exposed and transected. The sural nerve was also transected at its branch point from the sciatic and then freed from surrounding tissues and desheathed. In a mineral oil pool formed out of skin the nerve was divided into about 8 filaments and one was placed on bipolar hook electrodes for multi‐fiber recording. Action potentials were amplified and filtered (10 Hz ‐ 10 kHz) by a differential amplifier (DAM 50, WPI) and digitized by a data acquisition system (micro1401, Spike2. CED Ltd., UK). The receptive field area of the filament was mapped at the lateral edge of the foot by mechanical probing and pinching. There, a pair of stainless steel needles was inserted for electrical stimulation supramaximal for C‐fibers (2 ms, 10–20 V; DS2, Digitimer Ltd., UK). A filament was chosen if 1) responses could be evoked by pinching in the hairy skin, and 2) if Aδ‐ and C‐volleys could electrically be evoked. Only one filament was recorded per animal and hindpaw. Spontaneous neural activity was recorded for 5 min, then either 50 µl of saline were injected into the receptive skin area of the filament or one of four different formaldehyde concentrations in saline (see Figure 4), and recording was continued for 80 min. The mean spontaneous activity during the control period was subtracted from all recordings after intradermal injection, which were sequentially averaged in 5 min periods.

### 2.6 Stimulated CGRP release from mouse skin, peripheral nerve, and trachea

Hairy skin flaps of both mouse hindpaws were excised from the lower leg and foot and wrapped around acrylic glass rods with the corium side exposed (Kessler et al., 1999). The sciatic nerves were excised from their point of emergence out of the lumbar plexus to their trifurcation into tibial, sural and peroneal nerves, and the nerve trunk was desheathed by removing the epi‐ and perineurium (Sauer et al., 1999). The trachea was excised from the thyroid cartilage to the carina together with the two main bronchi, hemisected along the sagittal midline (Kichko & Reeh, 2009). The tissue samples were placed in carbogen‐gassed SIF inside a set of shaking baths for a washout period of 30 min. A temperature of 32°C for hindpaw skin and sciatic nerve and 37°C for trachea was maintained throughout the experiment. Following the washout period, all preparations were consecutively passed through a set of five glass tubes containing 700 μL SIF for skin flaps and 125 µL SIF for nerve or trachea, each incubation step lasting 5 min. The first two incubation steps were to determine basal CGRP release and its variability. The third incubation step assessed formaldehyde‐evoked CGRP release, using 0.4, 1.2, 4, 12, 40, 100 mM concentration on different tissue samples. The fourth and fifth incubation steps assessed recovery from the response. The CGRP content of the incubation fluids was measured using commercial enzyme immunoassay kits with a detection threshold of 5 pg/ml (Bertin Pharma, Montigny‐le‐Bretonneux, France). Formaldehyde solutions alone caused a concentration‐dependent distortion of the EIA’s CGRP calibration curve. To determine appropriate correction factors, solutions containing CGRP 200 pg/ml were supplemented with the experimental formaldehyde concentrations and determined at least threefold. Incubation fluids contaminated with formaldehyde were corrected by the determined correction factor: 0.86 for 0.12 mM, 0.84 for 0.4 mM, 0.89 for 1.2 mM, 1.12 for 4 mM, 1.38 for 12 mM, 2.24 for 40 mM, and 2.85 for 100 mM.

### 2.7 DRG cell culture

Dorsal root ganglia were harvested from adult C57BL/6J and congenic TRPA1^–/–^ mice. Ganglia were transferred to Dulbecco's modified Eagle's medium (Life Technologies, Germany) supplemented with gentamycin (50 µg/ml, Sigma‐Aldrich), and exposed to 2.2 U/ml collagenase and 1.5 U/ml protease (Sigma‐Aldrich, Germany) for one hour. The cells were dissociated with fire‐polished Pasteur pipettes and plated on glass cover slips coated with Poly‐D‐Lysine (200 µg/ml, Sigma‐Aldrich). DRG neurons were incubated in serum‐free TNB 100 medium supplemented with TNB 100 protein‐lipid complex (Biochrom, Berlin, Germany), penicillin and streptomycin (100 U/ml each, Life Technologies, Germany), and nerve growth factor (mouse NGF 2.5S, 100 ng/ml; Alomone Labs, Tel Aviv, Israel) at 37°C and 5% CO_2_. Electrophysiological recordings or calcium microfluorimetry were performed within 15–30 hours after dissociation.

### 2.8 Patch clamp recordings

Whole cell recordings were performed on small diameter DRG neurons. Membrane currents were acquired with an Axopatch 200B amplifier, controlled by pCLAMP 9 software (Axon Instruments/Molecular Devices, Sunnyvale, CA). Data were low‐pass filtered at 1 kHz and acquired at 2 kHz. Electrodes were pulled from borosilicate glass tubes (TW150F‐3; World Precision Instruments, Berlin, Germany) and heat‐polished to give a resistance of 2–3 MΩ. The standard extracellular solution (see above) was used, the internal solution contained (in mM) 140 KCl, 2 MgCl_2_, 5 EGTA and 10 HEPES, pH 7.4 was adjusted with KOH. For voltage clamp recordings, neurons were held at −60 mV. All recordings were performed at ~21°C room temperature. Solutions were applied with a gravity‐driven and software‐controlled common outlet perfusion system (Dittert et al., 1998).

### 2.9 Intracellular ratiometric calcium measurements

DRG neurons were loaded with the fluorescent calcium indicator dye Fura‐2‐AM 5 µM supplied with 0.02% pluronic F‐127 (Invitrogen Molecular Probes, Eugene, OR, USA) for 30 min. Fura‐2 was excited at 340 and 380 nm with 3 ms exposure time at 1 Hz, fluorescence at >490 nm was collected. Images were acquired with TillVision software from a peltier‐cooled slow‐scan CCD camera system with a PolyV monochromator (Till Photonics, Graefelfing, Germany) coupled to an inverted microscope. Background for both wavelengths was continuously recorded and subtracted before conversion to the F340/F380 fluorescence ratio. Cells were continuously superfused at a rate of 0.3 ml/min through a gravity driven system from a capillary positioned 100–250 µm from the cells with extracellular solution containing 1.25 mM CaCl_2_, resembling interstitial fluid calcium levels (Gilányi et al., 1988). The AUC of the ratio during application periods compared to control periods was used for analysis. KCl 60 mM was applied at the end of the experiment to discard nonresponsive cells and acquire a maximum response.

### 2.10 Data analysis

For CGRP release experiments, mean results in pg/ml are reported. For construction of the concentration‐response curves, the stimulated increase above baseline (delta CGRP, step three minus step two) was analyzed. Statistical comparisons were performed with Statistica (Statsoft, Tulsa, USA). Stimulated increases of CGRP release above baseline were tested with the nonparametric Wilcoxon matched pairs test. Experimental designs with separate groups and repeated measurements were tested by ANOVA. Post‐hoc comparisons against a single group were performed by Dunnett's t‐test, pair‐wise comparisons by Tukey's HSD. *p* < 0.05 was considered significant. A sigmoidal function was fitted to concentration‐response data using Origin (OriginLab Corporation, Northampton, USA). P values above 0.0001 are presented with three significant figures. Data are presented as mean (SD) or mean with 95% confidence interval of the mean.

## 3 RESULTS

### 3.1 In vivo pilot experiments

The pilot experiments were done in the anesthetized adult rat with single‐fiber recordings from the saphenous nerve and strictly intradermal formalin injections (2.5% of the saturated commercial solution = 308 mM formaldehyde) into or adjacent to receptive fields of identified low‐threshold mechanoreceptors and nociceptors of the hairy skin. The results are not reported in detail, because they are essentially identical to the findings previously published by two other groups applying the same methods (McCall et al., 1996; Puig & Sorkin, 1996). Briefly summarizing, the formalin injection into receptive fields evoked vivid discharge activity in each case, mostly beginning before the injection was completed and lasting up to 6 min, after which the receptive field under observation had regularly lost its responsiveness to mechanical, heat, cold, and electrical stimuli. Therefore, we injected Evans Blue‐stained formalin (5–15 µl) adjacent to the receptive field so that the injection bleb just reached its border (as in Puig & Sorkin, 1996). Again all fiber types (Aβ, Aδ and C; n = 12) were excited after a very variable delay due to diffusion, and only several of the small Aδ and C‐fibers ‘survived’ this procedure and partially retained responsiveness. A representative example is illustrated (Figure 1) and shows a decrescendo of the initial formalin response and, subsequently, a markedly facilitated radiant heat response of a typical ‘polymodal’, mechano‐heat sensitive C‐fiber (CMH), ~ 5 min after injection. Mechanosensitivity was lost at the side of injection but retained as before at the opposite side of the receptive field with a von Frey threshold of 22.6 mN. Thus, the most obvious effect of formalin in the usually high, fixative, concentration was to ‘kill’ the fully exposed nerve endings, a finding not unexpected. The lack of control in vivo over concentration and distribution of formaldehyde prompted us to perform most of the further experiments *in vitro* using isolated tissue preparations.

### 3.2 Concentration‐dependent acute effects of formaldehyde and follow‐up

**FIGURE 2:**
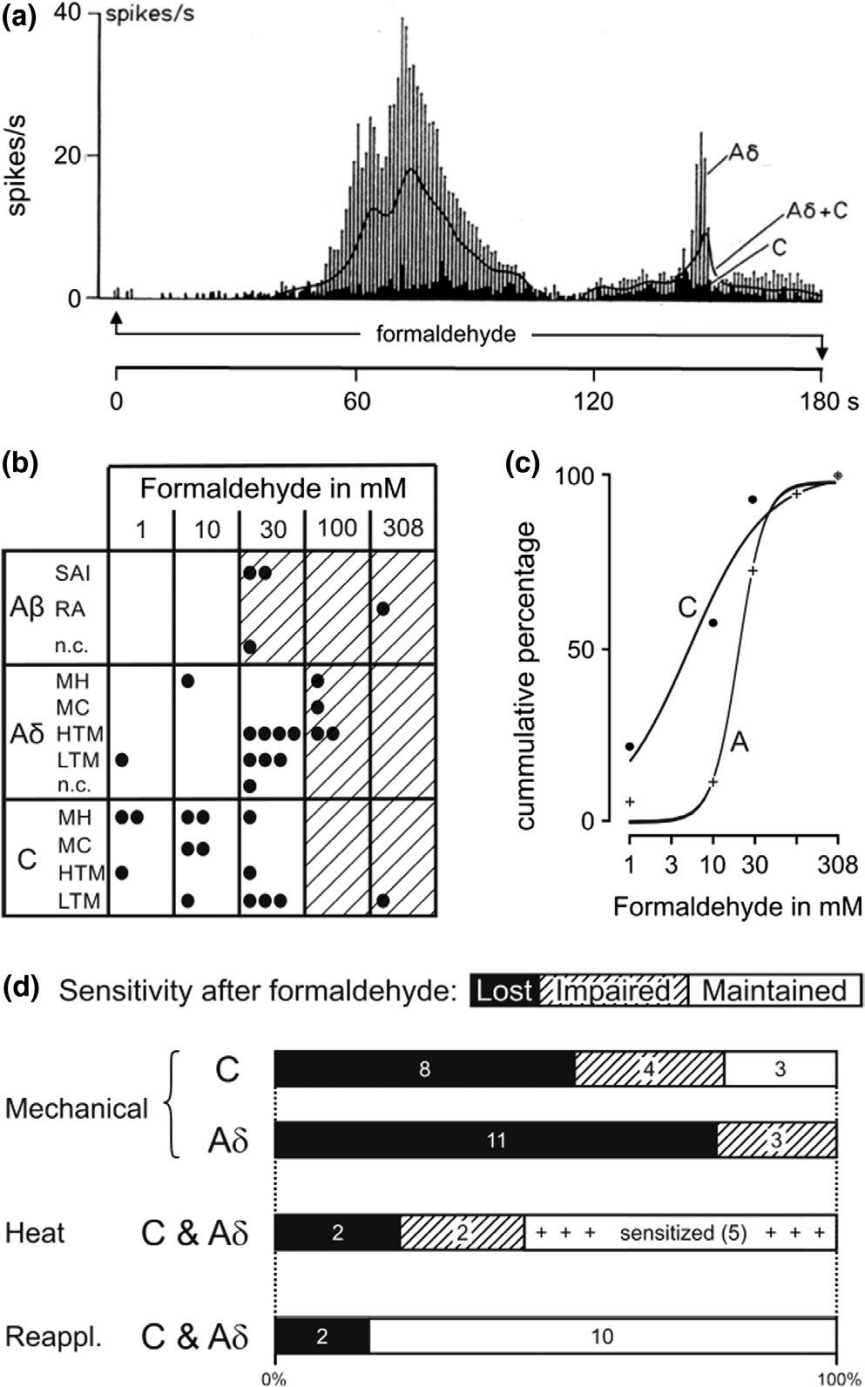
Excitation by formaldehyde and sensitivity changes of rat cutaneous single‐fibers *in vitro*. (a) Nociceptor activities during the first three minutes of formaldehyde application to receptive fields at threshold concentration (1, 10 or 30 mM); vertical bars 5 Aδ‐, black columns 5 C‐fibers, solid line average of both. (b) Formaldehyde threshold concentrations and fiber types in matrix form; each dot represents one individual fiber (n = 32), hatched fields indicate loss of thermal and mechanical sensitivity after transient excitation. (c) Cumulated responsiveness of C‐fibers (EC_50_ 5.2 mM), which were more sensitive to formaldehyde compared to A‐fibers (EC_50_ 24.3 mM). (d) Sensitivity changes of the C and Aδ‐fibers after exposure to formaldehyde threshold concentrations. Heat sensitivity was less vulnerable, even enhanced (“sensitized”), than mechanosensitivity; “impaired” means loss of responsiveness in part of the receptive field or marked increase of stimulus threshold. “Reappl.” applies to responsiveness upon a second formaldehyde application at higher than threshold concentration

The isolated rat skin‐nerve preparation was employed to determine the threshold concentration of formaldehyde to activate discharge in receptive fields of identified cutaneous nerve fibers (n = 32). Half of the fibers from the three main classes (Aβ, Aδ and C) were first exposed for 3 min to 0.1 mM formaldehyde and none was excited. After 3 min of wash‐out the next higher concentration was applied and so forth until the unit started firing action potentials. A representative sample of responses to formaldehyde threshold concentrations (1, 10 or 30 mM) was averaged and shows (Figure 2a) that Aδ‐fibers, not surprisingly, contribute a lot more to nociceptive discharge than an equal number of C‐fibers which, however, are more numerous in the skin. The majority of C and two Aδ‐fibers with their unmyelinated endings were sensitive to 1 and 10 mM formaldehyde, while the remaining small‐fiber units were only excited at 30 mM (Figure 2b). The EC_50_ of the C‐fibers was 5.2 mM (CI 1.9–7.1), which is lower than the 21.2 mM (CI 18.8–23.6) of the myelinated fibers, whereby only about 19% of fibers required more than 30 mM formaldehyde to get excited (Figure 2c). Following exposure to their individual threshold concentration, most units were re‐tested for mechanical and heat sensitivity. The four Aβ‐fibers had lost their mechanosensitivity at 30 mM, and all units requiring higher formaldehyde concentrations to get excited were completely desensitized to thermal and mechanical stimulation (hatched area in Figure 2b). At lower concentrations, a loss of mechanosensitivity after formaldehyde occurred much more frequently than a loss of (pre‐existing) heat sensitivity (χ^2^ = 4.7, *p* = 0.0310; Figure 2d). Even more, 5/9 polymodal nociceptors were clearly sensitized (like in Figure 1c) to heat after formaldehyde, although two of them (Aδ and C) had lost their mechanosensitivity. In addition, a second formaldehyde application at the next higher concentration was effective in 10/12 C and Aδ‐fibers 5–30 min after the first response, mostly evoking greater discharge activity than before. In conclusion, all cutaneous nerve fibers can be excited by a sufficiently high formaldehyde concentration, but unmyelinated nerve endings seem to be more sensitive than myelinated fibers; mechanosensitivity appears more vulnerable than chemical or heat sensitivity, while the latter can even be enhanced after 3 min formaldehyde exposure at threshold concentration.

### 3.3 The second phase of small fiber discharge

**FIGURE 3:**
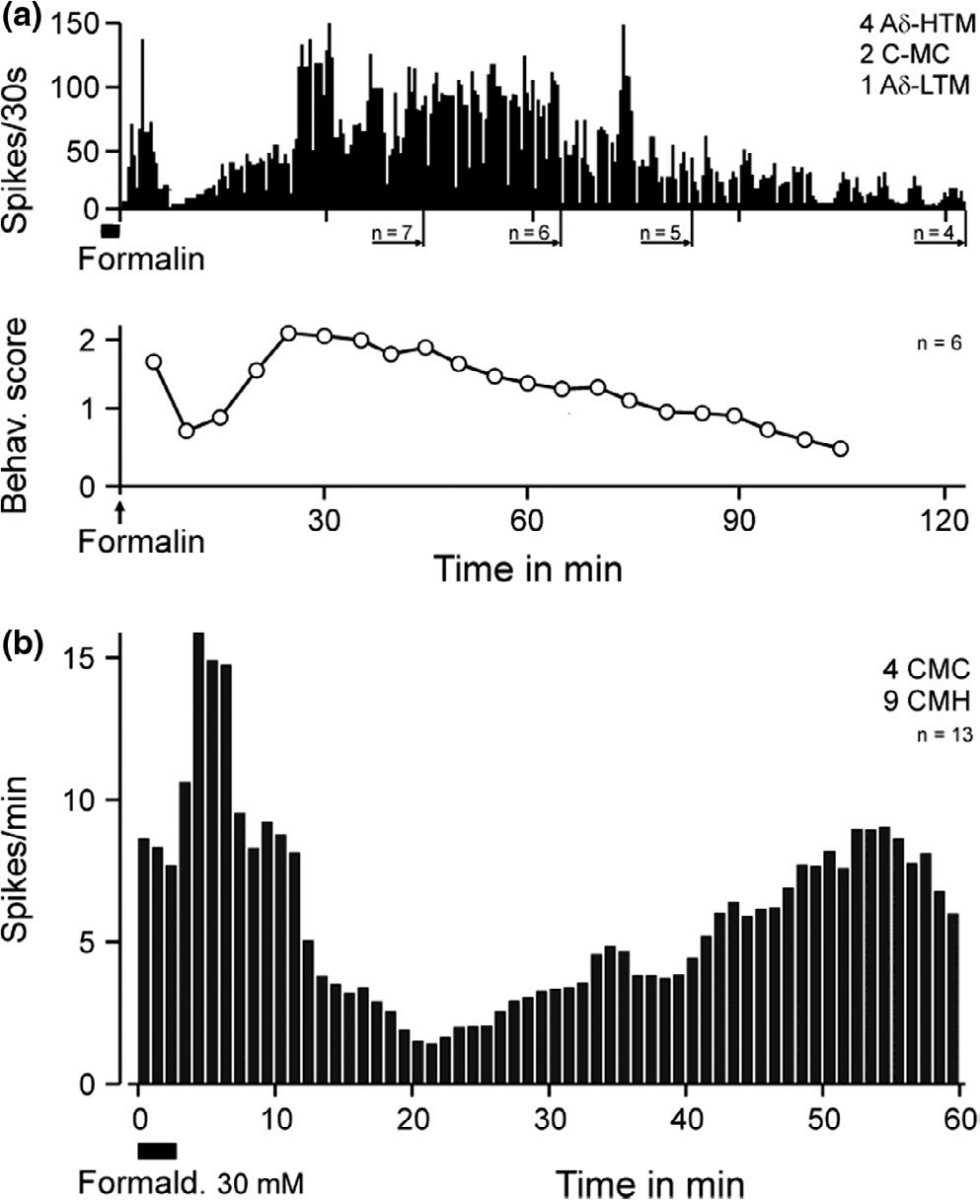
Two phases of rat single‐fiber activity *in vitro* and behavioral correlate. (a) Averaged fiber activities of a sample of receptive fields exposed for 3 min to formaldehyde 30 or 100 mM; falling number of units due to action potentials sinking into background noise. Lower panel (after Kocher, 1988) showing mean time course of pain‐related behaviors (shaking, licking, biting) after formalin (2.5%) injection in Wistar rats; cross‐correlation in 5 min bins provided R = 0.67 (*p* = 0.0185, product‐momentum correlation). (b) Averaged rat fiber activity starting with uniform formaldehyde 30 mM application to receptive fields of only polymodal C‐fibers

Already after the formalin injections adjacent to receptive fields *in vivo* we observed a late second phase of seemingly spontaneous discharge activity in 2/7 C‐fibers, confirming the two published reports that previously described this phenomenon (McCall et al., 1996; Puig & Sorkin, 1996). Nonetheless, it was surprising that a second discharge phase after formaldehyde wash‐out also occurred in isolated rat skin, in the absence of blood supply and inflammation (Figure 3). The four Aβ‐units (of Figure 2B) remained silent after their initial transient formaldehyde response and did not regain mechanosensitivity and/or electrical excitability within 30 min; they also did not respond to a second formaldehyde challenge at higher concentration. However, in a subset of 15 C and Aδ‐fibers (out of the Figure 2b sample) which could be followed‐up, 8/15 units developed surging discharge activity after a variable period of quiescence following their initial response, which had required either 30 mM or 100 mM formaldehyde. The other 7/15 C and Aδ‐units had responded to lower (threshold) concentrations or 30 mM but thereafter remained silent for 30 min. We assumed that the dose of formaldehyde deposited in the skin had not been sufficient to induce (by diffusion) a second phase of discharge. Therefore, these particular fibers received a second formaldehyde challenge for 3 min at 30 mM or 100 mM, respectively, and all of them showed a prompt transient response, mostly greater than before, followed by quiescence. In both groups of these small fibers (n = 15) the quiescence periods lasted for 4–13 min, hardly interrupted by single spikes (up to 6). Then also the second, twice exposed, group of units (7/15) developed surging discharge activity in the absence of overt stimulation. The second phase of firing lasted longer than two hours in individual cases, but only few fibers could be followed up that long due to run‐down of the action potential amplitude. A sample of initially seven units standing at least 40 min after formaldehyde application was averaged and shows the hangover of the first discharge phase, the quiescent interphase, and the second phase with a decreasing number of fibers averaged (Figure 3a upper panel); four of these units had lost their mechanosensitivity after the first discharge phase. This sample of units definitely comprised one slowly adapting low‐threshold (~ 1 mN von Frey) mechanoreceptive Aδ‐fiber (standing 120 min) that would not be considered a nociceptor and did not regain mechanosensitivity. Only few studies have assessed formalin pain‐related behavior for longer than one hour; a particular one is chosen from the literature to illustrate the good correlation of time courses (Figure 3a lower panel, from (Kocher, 1988). Discharge activity and behavioral score were cross‐correlated in 5 min bins and provided R = 0.66 (*p* = 0.0185, product‐momentum correlation).

Aδ‐fibers were overrepresented in the sample above, formaldehyde was partly applied twice at unequal concentrations (30 or 100 mM), and the first 3 min of the recording were missing (in Figure 3a). For these inconsistencies, we later repeated the study on the rat skin‐nerve preparation, applying uniformly 30 mM formaldehyde for 3 min to receptive fields of only polymodal C‐fibers and averaging only units that were able to stand 60 min of recording (Figure 3b). The outcome, in principle, was the same as described above: two phases of discharge activity, a nadir in between, though less distinct and more delayed, and lower discharge rates as expected from C‐fibers, but all units showed a second phase of firing. During the quiescent interphase or later when discharge was fading out electrical excitability was re‐tested at several spots inside the metal ring (and receptive field) area where formaldehyde had been applied. Six of the 13 C‐fibers could be activated by cathodic stimuli of up to 90 V at 1 ms duration. In conclusion of the rat skin experiments, all C and Aδ but no Aβ‐fibers exposed in their receptive fields to a minimum of 30 mM formaldehyde developed a second phase of discharge activity after a transient first phase and a quiescent interphase.

### 3.4 Biphasic discharge in a peripheral nerve in vivo

**FIGURE 4:**
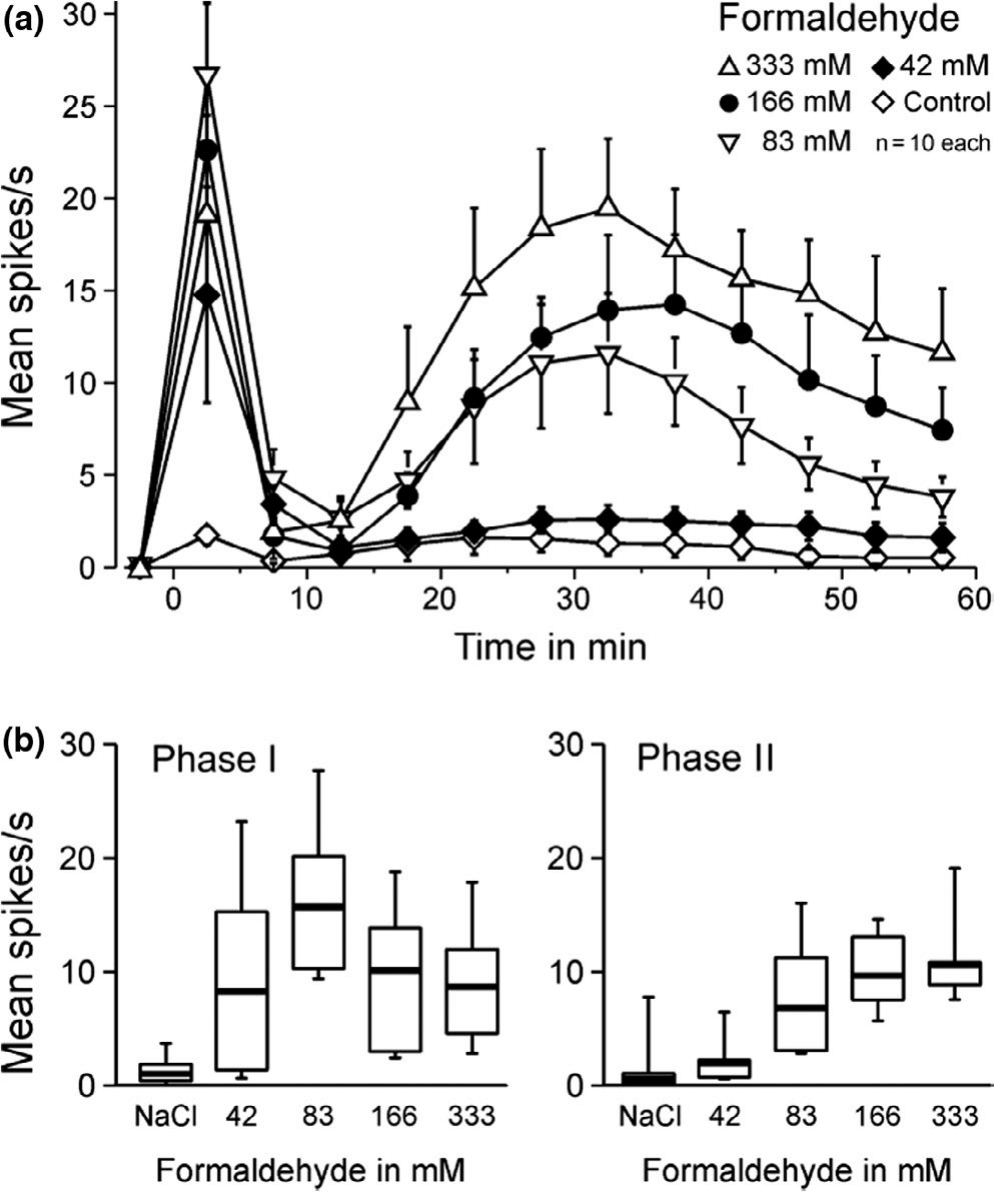
Formaldehyde concentration dependence *in vivo* of multi‐unit activity in sural nerve filaments of anesthetized Sprague‐Dawley rats. (a) Formaldehyde 42–333 mM was injected into the lateral foot edge evoking prolonged biphasic mass activity in the sural nerve filaments which was dominated in phase 1 by tall spikes missing in phase 2; the second phase appeared only above 42 mM. Data are mean ± SEM (b) Concentration‐dependence of both phases. The lowest concentration with a significant difference to the saline control (NaCl) was formaldehyde 83 mM. In the box plots the median is indicated by thick lines, boxes indicate 25–75 percentiles and whiskers 10–90 percentiles

Single‐fiber recordings are necessarily eclectic ‐ determined by mechanical search stimulation in our case ‐ and may not truly represent the whole of a neuronal population responding to a gross formalin injection. We therefore employed multi‐fiber recordings *in vivo* from rat sural nerve filaments thin enough to provide C‐fiber action potentials exceeding the noise level. The window discriminator was adjusted as to capture each spike above noise, including A and C‐fibers. The sural nerve innervates the lateral edge of the hindpaw footpad, where formalin was intradermally injected in various concentrations (Figure 4a). During the first phase of evoked discharge activity, high amplitude spikes probably originating from Aβ‐fibers were visible. These were missing during the second phase that was dominated by smaller spikes most likely from Aδ and C‐fibers. The concentration‐response relationship regarding the first phase appeared inversely U‐shaped with a maximum at 83 mM formaldehyde, while for the second phase it showed an ascending slope with a tendency towards saturation (Figure 4b). The lowest concentration tested (42 mM) caused a first phase of discharge but no second phase, just like in our published behavioral results from injection of 39 mM formaldehyde (Fischer et al., 2014). In conclusion, the primary afferent population response is most suggestively congruent with the behavioral response in respect to both shape and magnitude of both discharge phases.

### 3.5 Concentration‐dependent differential nerve block in vitro

**FIGURE 5:**
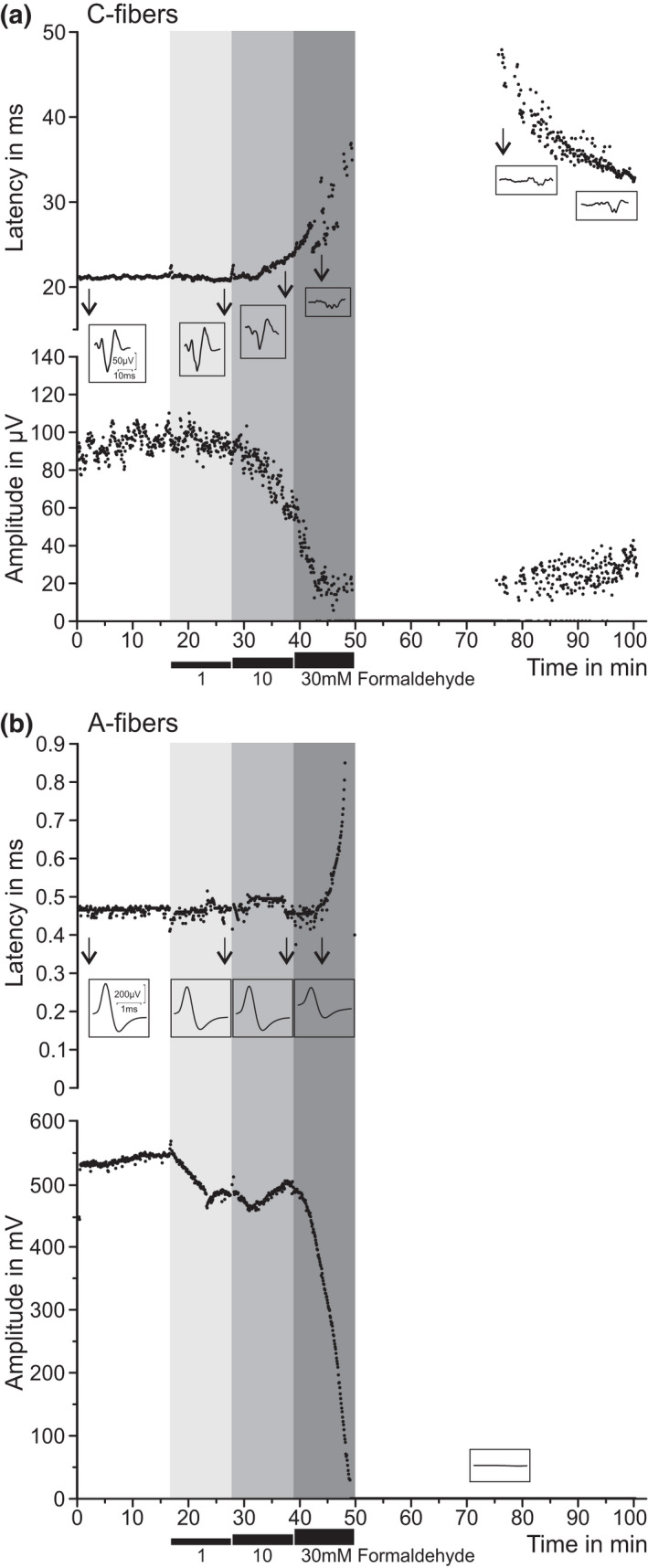
Formaldehyde > 10 mM obliterates nerve conduction in A and C‐fibers *in vitro*. Isolated desheathed rat sciatic nerves were superfused with increasing concentrations of formaldehyde (1–30 mM) and CAPs of C‐ and A‐fibers evoked by supramaximal electrical stimulation were recorded; three experiments with equal outcome. Insets show single CAPs registered at critical time points. (a) C‐fiber CAPs showed a progressive increase in latency (upper panel) and decrease in amplitude (lower panel) upon 10 mM formaldehyde, leading to complete conduction block at 30 mM with rudimentary recovery. (b) In the same nerve, A‐fibers showed slightly less sensitivity to formaldehyde and no recovery of the conduction block

To fill the anatomical gap between the single and multi‐fiber recordings here and the previously published cellular results (Fischer et al., 2014), we also recorded electrically evoked compound action potentials (CAPs) from the isolated rat sciatic nerve while applying incremental formaldehyde concentrations comparable to the threshold concentrations for single‐fiber activation (Figure 5). These experiments (n = 3) confirmed the slightly higher sensitivity but greater tolerance to formaldehyde of C versus A‐fibers seen with single‐fiber recordings. During 10 mM formaldehyde superfusion the amplitude of the C‐CAP decreased while the latency increased, both parameters changing perfectly in parallel and most similar to the effects of a local anesthetic (Brenneis et al., 2014); total block occurred at 30 mM formaldehyde. The A‐CAP shrank a little at 1 mM and 10 mM without latency change and required 30 mM formaldehyde to vanish but did not recover from total block within 50 min of wash‐out, in contrast to C‐fibers which partly returned to function after 25 min with a small and long latent C‐CAP (~ 0.33 m/s).

In conclusion, although 30 mM formaldehyde is just a tenth of a usually injected formalin concentration (2.5%) in vivo, it was sufficient to irreversibly block all A‐fibers and let ‘survive’ only a small fraction of slow C‐fibers in peripheral nerve.

### 3.6 TRPA1‐dependent responses in cultured DRG neurons

**FIGURE 6:**
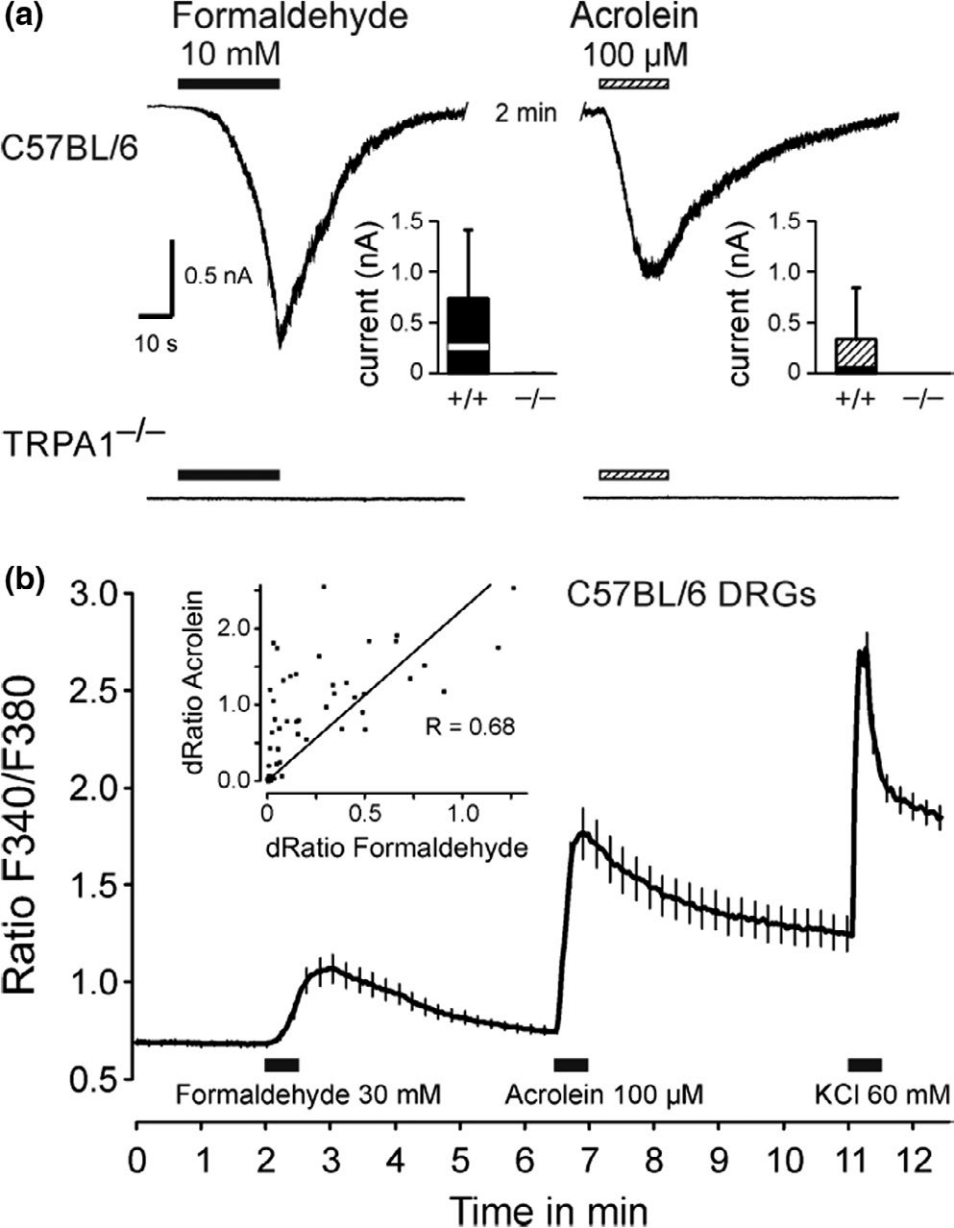
TRPA1‐dependent formaldehyde responses in cultured mouse DRG neurons. (a) Representative current traces of C57BL/6J and TRPA1‐knockout neurons upon applications of formaldehyde 10 mM or acrolein 100 µM. Cells were held at –60 mV and compounds were applied for a period of 30–40 s at an interval of two minutes. The insets show current amplitudes evoked by formaldehyde and acrolein. Formaldehyde evoked currents in 10 of 16 and acrolein in 8 of 16 C57BL/6 neurons. In contrast, 1 of 31 TRPA1 knockout neurons was activated by formaldehyde, none by acrolein. In the box plots boxes indicate 25–75 percentiles and whiskers 10–90 percentiles. (b) Formaldehyde and acrolein reversibly increase intracellular calcium levels. The main panel shows the time course of intracellular calcium; solutions were applied for 30 s (n = 105, vertical lines indicate standard errors). Inset: The increases in calcium evoked by formaldehyde and by acrolein in these neurons were correlated

The discovery that the almost universal chemoreceptor and cation channel TRPA1 is a mediator of the formaldehyde effect provided a plausible explanation for nociceptor excitation and pain‐related behavior (Macpherson et al., 2007; McNamara et al., 2007). In our hands, whole‐cell current‐clamp recordings from small and medium sized C57BL/6J mouse DRG neurons showed a formaldehyde‐evoked depolarization that concentration‐dependently affected increasing numbers of neurons (100% at 40 mM, EC_50_ 10 mM); at 40 mM the depolarization amounted to about 20 mV and lasted for about 30 s (like in Figure 2d of Fischer et al., 2014). Voltage‐clamp recordings confirmed the TRPA1‐dependance for a relatively low formaldehyde concentration (Figure 6a). In wildtype cells formaldehyde 10 mM evoked inward currents in 10 of 16 neurons, 8 of these also responded to the TRPA1 agonist acrolein. However, 0 of 31 DRG neurons from TRPA1‐deleted mice showed any inward current upon stimulation with either formaldehyde or acrolein.

Cross‐sensitivity of neurons to different specific agonists is better to be judged with larger cell counts as achievable with calcium imaging (Figure 6b). Formaldehyde 10 mM activated 26% of wildtype DRG cells responsive to KCl but none from TRPA1 knockout (data not shown). At formaldehyde 30 mM the magnitude of calcium transients was positively correlated with those elicited by acrolein, a typical electrophilic TRPA1 agonist (R = 0.68, *p* < 0.0001, n = 105). Although TRPA1 appears as a predominant mediator of neuronal formaldehyde effects, these results leave room for other mechanisms of evoked intracellular calcium rises (Fischer et al., 2015).

### 3.7 Formaldehyde‐induced CGRP release from isolated tissues

**FIGURE 7:**
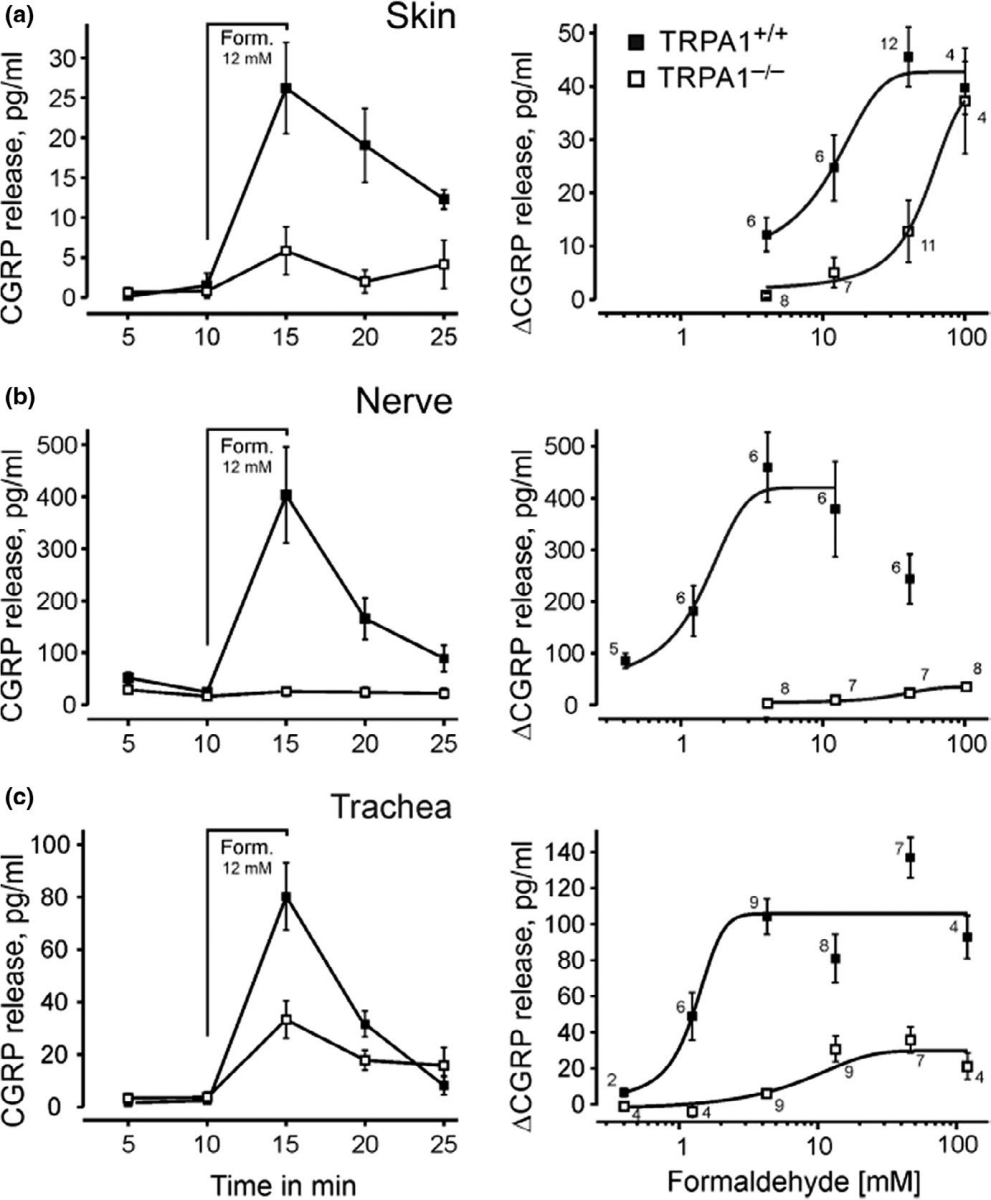
Formaldehyde‐evoked iCGRP release from isolated tissues of TRPA1^–/–^ and WT littermates. Time course of CGRP release in (a) hindpaw hairy skin, (b) desheathed sciatic nerve, and (c) trachea; the five‐minute stimulation period with formaldehyde (Form. 12 mM) is indicated. Right panels summarize the formaldehyde concentration‐response curves; sigmoidal functions are fitted to the increases in CGRP release. The numbers of identical experiments are given for each data point. Means and standard errors are visualized

Another highly integrative index of neuronal activation results from measuring stimulated CGRP release from isolated intact tissues, here taken from mouse TRPA1^–/–^ and wildtype littermates. CGRP is contained in the vast majority of TRPV1 and TRPA1 co‐expressing sensory neurons and released upon calcium influx (Gebhardt et al., 2020; Ushio et al., 2013). This also applies to axons in peripheral nerves, which carry the functional receptor‐channels all along their axolemma and perform calcium‐dependent vesicular exocytosis of neuropeptides (Bernardini et al., 2004; Weller et al., 2011). In fact, the mouse sciatic nerve was a particularly productive preparation, releasing enormous amounts of CGRP upon formaldehyde stimulation in a concentration‐dependent way (EC_50_ = 1.5 mM, CI 0.5–2.5 mM) with decreasing release at concentrations higher than 4 mM and almost completely dependent on TRPA1 expression up to 100 mM (Figure 7b). The trachea is the only organ that in humans is habitually exposed to exogenous formaldehyde when cigarette smoke is inhaled (Pouli et al., 2003). The mouse trachea showed about the same concentration‐response relationship of formaldehyde as the nerve but more unspecific, TRPA1‐independent effects (Figure 7c). The EC_50_ in the skin was found about one decade higher at 10 mM (CI 3–17 mM), however the unspecific effect in TRPA1 null mutants at 100 mM formaldehyde reached the magnitude of the response in wildtype mice (Figure 7a). The possible involvement of TRPV1 (Tian et al., 2009) in formaldehyde‐induced CGRP release was only tested in the trachea and at 12 mM concentration; TRPV1 null mutants showed slightly larger responses than TRPV1 or TRPA1 wildtypes (data not shown). In conclusion, TRPA1 accounts for most of the formaldehyde‐evoked CGRP release in deep tissues, while in the skin at higher concentration (> 30 mM) this formaldehyde effect is the same whether or not TRPA1 is expressed.

### 3.8 Biphasic single‐fiber discharge in TRPA1 wildtypes and knockouts

**FIGURE 8:**
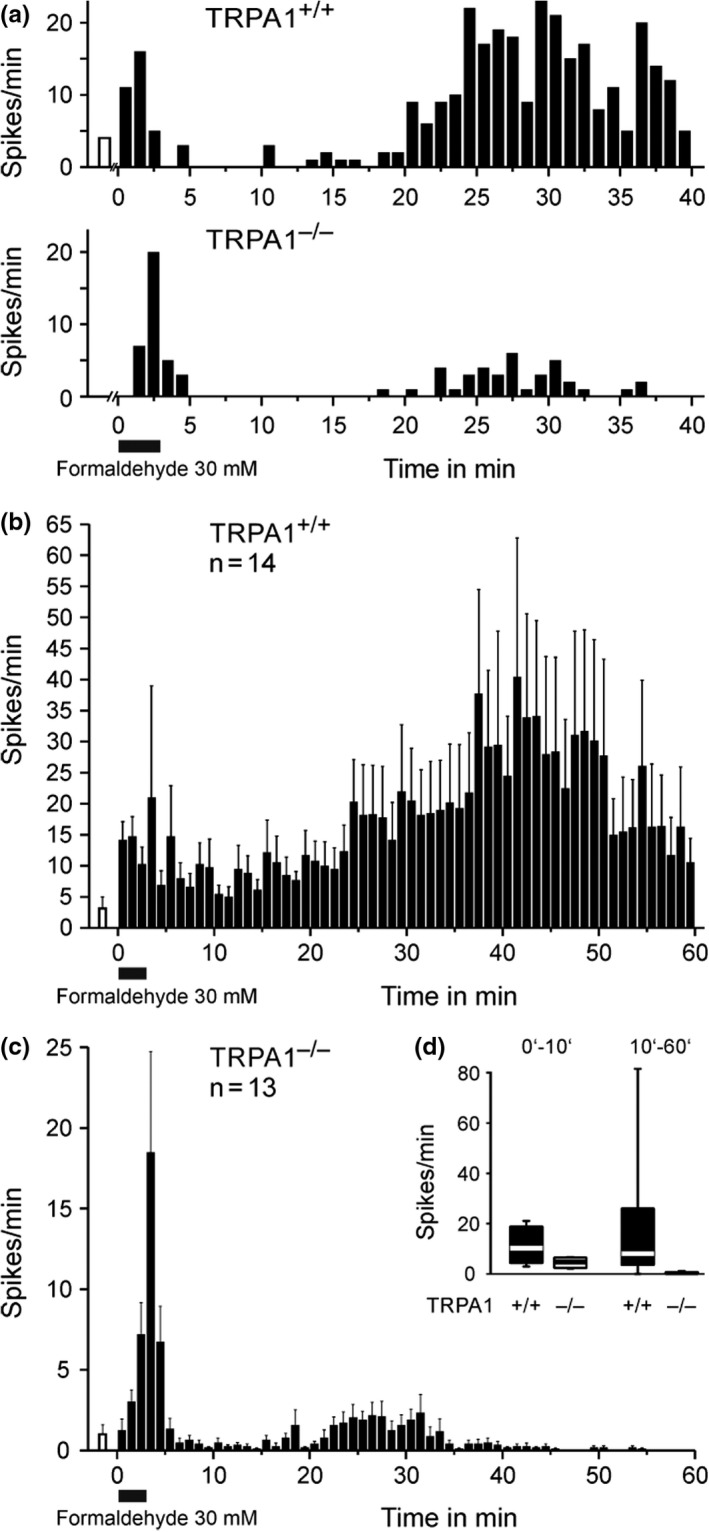
Biphasic single‐fiber activity in TRPA1^–/–^ and WT littermates. (a) Specimen demonstrating the biphasic discharge pattern upon formaldehyde 30 mM/3 min application to receptive fields of a TRPA1 wildtype and knockout C‐MH fiber. (b) Mean discharge rates for all nociceptive and non‐nociceptive C and Aδ‐fibers (see text) recorded in TRPA1 wildtype and (c) TRPA1 knockout animals; note the different Y‐scales. The open columns before formaldehyde application demonstrate the mean ongoing activity after mechanical and thermal characterization of fibers. Data are mean ± SEM. (d) Mean discharge rates within the minutes 0–10 and 10–60 after formaldehyde of all fibers depicted in B and C (ANOVA main effect for genotype *p* = 0.00968). In the box plots the median is indicated by thick lines, boxes indicate 25–75 percentiles and whiskers 10–90 percentiles

When TRPA1^–/–^ were injected with only 60 mM formaldehyde solution, they showed a substantial attenuation of the usual licking/lifting behavior but no abolition in both phases of the formalin test (McNamara et al., 2007). Precisely this was recapitulated in our single‐fiber recordings from the isolated mouse skin‐nerve preparation (Figure 8). Mostly ‘polymodal’ (C‐MH) units, one C‐MC and two slowly adapting low‐threshold mechanosensitive C and Aδ‐fibers were recorded in the wildtype group of mice, and formaldehyde was applied for 3 min at 30 mM concentration as before in the rat. From the TRPA1^–/–^ animals eight polymodal and one high‐threshold mechanosensitive C‐unit and four high‐threshold mechanosensitive Aδ‐nociceptors were averaged in the peri‐stimulus time histogram. All fibers (n = 27) responded to formaldehyde, all showed a quiescent interphase and a second phase of discharge activity, more pronounced in TRPA1^+/+^ and much less in TRPA1^–/–^ (Figure 8). The quantitative difference between the genotypes was less marked during phase 1 (Figure 8d), the more obvious during the interphase and in phase 2, which also was clearly shortened, fading already after 35 min (Figure 8a and c). The activity in TRPA1^–/–^ in phase 1 was 33% and in phase 2 only 3% of the TRPA1^+/+^ fibers. A significant interaction phase*genotype indicates that the second phase is more TRPA1 dependent than the first phase (ANOVA, F_(1, 25)_ = 4.3, *p* = 0.0490), with a clear main effect of the genotype (*p* = 0.00968). Thus, TRPA1 is the predominant but not the only driving force at 30 mM formaldehyde, in particular in the second phase of small fiber discharge activity which, notably, includes biphasic firing of non‐nociceptive small fibers.

## 4 DISCUSSION

In order to question the widespread assumption that the pain‐related behaviors in the second phase of the formalin test in rodents requires inflammatory or spinal sensitization, we are reporting data largely gained from isolated superfused tissue preparations without the spinal cord. Only pilot experiments with rat single‐fibers and sural nerve filament recordings were done *in vivo* and confirm previous findings that the usually high fixative formaldehyde concentrations transiently excite and then block every sensory nerve ending in the injected skin patch, followed only in small Aδ‐ and C‐fibers by a quiescent interphase and a sustained second phase of discharge, dose‐dependent with regard to magnitude and duration (McCall et al., 1996; Puig & Sorkin, 1996). However, these *in vivo* experiments could not exclude inflammatory processes to induce and sustain the second phase activity.

### 4.1 “Anesthesia dolorosa”

In the absence of blood supply, thus *in vitro*, the concentration of formaldehyde and time of exposure to characterized receptive fields of cutaneous single‐fibers could be controlled. This revealed that the unmyelinated nerve terminals of sensory Aδ and C‐fibers are more sensitive to transient excitation by formaldehyde (1–10 mM) than the myelinated Aβ‐fibers of low‐threshold mechanoreceptors, with an overlap between the fiber classes at 30 mM threshold concentration. This was also the concentration at which the first A‐fibers lost their sensitivity and (electrical) excitability after 3 min of exposure. To get completely defunctionalized, the other A and C‐fibers required 100 mM or, rarely, 308 mM formaldehyde which is the concentration injected in many behavioral studies (2.5% of the 37% commercial formalin solution saturated with formaldehyde). Thus, one can safely assume that the usually injected concentrations (1–10%) of the fixative render the affected skin patch anesthetic, probably for a long time. The formalin test is *quasi* a model of ‘anesthesia dolorosa’ ‐ in semantic, not clinical sense. In fact, a complete block of the rat sciatic nerve was achieved with only 30 mM formaldehyde, and only a few very slow‐conducting C‐fibers recovered after 25 min of wash‐out (Figure 5). Our previous patch‐clamp studies on mouse DRG neurons, applying 30 mM formaldehyde, found a transient depolarization with a brief decrescendo burst of spikes that turned into a profound hyperpolarization within one minute, accompanied by an equally profound loss of stimulated peak sodium inward currents (Fischer et al., 2014). Both silencing effects were prevented by a high, but not low, concentration of tetrodotoxin (TTX 100 versus 0.3 µM). This indicated that the relatively TTX‐resistant voltage‐gated sodium channels Na_V_1.8 and Na_V_1.9 were blocked by formaldehyde, the former shaping the action potential of small fiber neurons for the most part, the latter largely responsible for the persistent sodium current that keeps the resting membrane potential relatively depolarized and the neurons excitable (Lolignier et al., 2011; Matson et al., 2015).

### 4.2 Loss of mechanosensitivity

After application of low but excitatory formaldehyde threshold concentrations to cutaneous receptive fields, all A‐fibers and the vast majority of small fibers had lost all or part of their mechanosensitivity, while pre‐existing heat sensitivity was largely retained, even enhanced in several units (Figure 2d). The sensory transduction of mechanical stimuli in umyelinated nerve endings has not yet been fully clarified, but an interaction with keratinocytes and/or mechanosensitive Schwann cell processes may play an essential role (Abdo et al., 2019; Moehring et al., 2018). Differentiated keratinocytes respond to mechanical stimuli with a prolonged calcium influx, and this leads to release of ATP which seems to transmit a, at least sensitizing, signal to apposed nerve endings (Goto et al., 2010; Moehring et al., 2018; Sadler et al., 2020). We have recently shown that primary mouse keratinocytes respond to formaldehyde (EC_50_ 29 mM) with intracellular calcium increase caused by an efficient block of SERCA in their endoplasmic reticulum (Fischer et al., 2015). These interrelationships provide sufficient points of attack for formaldehyde to effect the posttranslational modifications of involved proteins that cause impairment or extinction of mechanosensitivity. Moreover, the so far identified mechanotransducing ion channels in nociceptors, TRPA1 and PIEZO2, are assumed to interact with cytoskeleton and extracellular matrix proteins or to be gated by force‐from‐lipid (Hill & Bautista, 2020; Lennertz et al., 2012; Moparthi & Zygmunt, 2020; Murthy et al., 2018; Startek et al., 2021). This provides further points of attack for formaldehyde to modify and cross‐link proteins, reduce membrane and tissue compliance, and so to impair mechanosensitivity.

### 4.3 Sensitization to heat

Mechanosensitivity seems to be restricted to the very nerve endings in the skin, fibers of passage in (healthy) peripheral nerves do not respond to adequate mechanical stimuli. Quite in contrast, nociceptive nerve fibers do show axonal heat sensitivity all along peripheral nerves, just like their individual terminals (Hoffmann et al., 2008, 2009), and this may explain why mechanosensitivity was mostly lost after formaldehyde (threshold concentration) while heat sensitivity was often retained, even enhanced, in the same polymodal nociceptors (Figure 2d). Axonal heat sensitivity is due to the even distribution of heat‐activated chemosensory receptor‐channels all along the nociceptive neuron, including the trias of essential heat transducers, TRPV1, TRPM3, and TRPA1 (Bernardini et al., 2004; Hoffmann et al., 2013; Moparthi et al., 2016; Sauer et al., 1999; Sinica et al., 2019; Vandewauw et al., 2018; Weller et al., 2011). TRPA1 is the first‐ranking target of formaldehyde evoking calcium influx through the channel in nociceptive neurons with an EC_50_ of 2.5 mM (Fischer et al., 2014; Macpherson et al., 2007; McNamara et al., 2007). Cytosolic calcium elevation is a dependable way to enhance noxious heat responses of DRG cell bodies and cutaneous nerve terminals (Guenther et al., 1999). Thus, sensitization to heat by an excitatory threshold concentration of formaldehyde, as observed, is a plausible effect, as the concentration just outside the application chamber in our skin‐nerve experiments may not be as high as to block the nerves but high enough to activate TRPA1 and enhance axonal heat sensitivity. This logic may also apply to the retained responsiveness of many axons to a second formaldehyde exposure, as observed (Figure 2d).

### 4.4 Concentration‐response relationships

Formaldehyde in aqueous solution diffuses rapidly in its hydrated form methanediol (48 g/mol, also called methylene glycol) and is in equilibrium with traces of formaldehyde gas (30 g/mol) (Fischer et al., 2014; Fox et al., 1985). The first and second phases of formalin pain‐related behavior in rodents are linked to each other by the partly parallel course of their formaldehyde dose‐response curves, which suggests a common cause of the excitatory effect, i.e. the rapid lateral diffusion in the skin along excitable nerve fibers. Two differences between the dose‐response curves do not contradict the theory: First, the first phase shows an inverted U‐shaped dose‐response (maximum 83 mM) while the second phase steadily climbs to high concentrations (Figure 4 and (Fischer et al., 2014)). A plausible reason for the inverted U is that higher formaldehyde concentrations, as in the injection bleb, block the nerve fibers faster and shorten the first discharge phase (Fischer et al., 2014). This, however, does not prevent the deposited formaldehyde to diffuse out of the blocked skin area and reach still excitable parts of the fibers, evoking a second phase of discharge activity. This diffusion takes some time, contributing to the quiescence in the interphase, and it leads to dilution of the spreading formaldehyde (i.a. by the plasma extravasation around the injection bleb). This may explain the second difference between the dose‐response curves which is the higher threshold concentration of formaldehyde to induce a second than first phase *in vivo*, 39 mM versus 19 mM in mice and 83 mM versus 42 mM in the thicker rat skin (Fischer et al., 2014), considering allometric scaling (and Figure 4 in (Wei et al., 2017). For a long time formaldehyde (CH_2_O) was the one and only chemical among the many algogenic, pain‐inducing, substances injected into rodent paws which evoked biphasic nocifensive behavior. However, in 2016 methylglyoxal (C_3_H_4_O_2,_ 72.06 g/mol, IUPAC: 2‐ketopropanal, also called acetylformaldehyde), was introduced as a biphasic algogen, again requiring a higher concentration to induce a second phase of pain after a quiescent interphase (Huang et al., 2016). This endogenous compound, a cytotoxic by‐product of glycolysis, was known before as contributing to the painful symptoms of experimental diabetes in rodents (‘diabetic neuropathy’) by accumulating in insulin‐independent cell types such as neurons (Bierhaus et al., 2012). And, methylglyoxal had been identified as an electrophilic TRPA1 agonist, binding to and posttranslationally modifying intracellular cysteine and lysine residues of the channel protein, just like formaldehyde (Andersson et al., 2013; Eberhardt et al., 2012). Accordingly, a TRPA1 antagonist (A‐967079) dose‐dependently reduced the second phase of methylglyoxal‐induced pain (Huang et al., 2016).

### 4.5 The second phase

In our single‐fiber recordings 1–10 mM formaldehyde was sufficient to transiently excite a considerable number of only small fibers, but 30 mM were required to regularly see a second phase of discharge in rat as well as mouse skin *in vitro*. Although all fibers of all classes were initially excited by formaldehyde, only all Aδ and C‐fibers, their endings lacking myelin sheaths, but not a single myelinated Aβ‐fiber developed a second discharge phase. However, not all of these small fibers were obvious nociceptors; three of our four different single‐fiber collections contained up to eight low‐threshold slowly adapting mechanoreceptive (LTM) units showing second phase discharge. This observation is in agreement with recent publications from Aziz Moqrich's laboratory; they had introduced a new biomarker for cutaneous sensory C‐fibers, a Gα_i_‐coupled protein named GINIP that marks two known neuronal subpopulations, the MrgprD‐expressing intraepidermal nociceptors involved in itching and the LTM C‐fibers, both non‐peptidergic neurons (Gaillard et al., 2014). When GINIP^+^ neurons were ablated by genetical engineering, the first phase of the formalin test (2%) was reduced, as was the touch sensitivity of the mice, and the second phase was almost, but not quite, abolished (Urien et al., 2017). However, the isolated genetical ablation of the large neuronal MrgprD^+^ subpopulation had previously been reported not to take any influence on the formalin test (Shields et al., 2010). Together these findings grant the LTM C‐fibers an unexpected role in, at least formalin‐induced, nociception and pain, as was first suggested for the mechanical hypersensitivity caused by injury and recently also for neuropathic cold allodynia (François et al., 2015; Seal et al., 2009).

### 4.6 Peptidergic neurons

Although non‐peptidergic neurons obviously contribute to the second (and less to the first) phase of the formalin test, formaldehyde was able to stimulate release of CGRP from our *ex vivo* tissue preparations in a concentration and TRPA1‐dependent manner. The sciatic nerve axons, in particular, yielded enormous amounts of stimulated neuropeptide release and almost none if TRPA1 was deleted (Figure 7). A large proportion of mouse DRG neurons with C and Aδ‐fibers label positive for CGRP, and all of them co‐express the TTX resistant Na_V_1.8 sodium channel (Patil et al., 2018). After ablation of the Na_V_1.8‐expressing subpopulation of neurons, the first phase of the formalin test remains unchanged but the second phase is almost abolished (Abrahamsen et al., 2008). Also the pharmacological block of Na_V_1.8 (by A‐803467) is able to abolish the second but not first phase of pain, in this case induced by the other biphasic algogen methylglyoxal (Huang et al., 2016).

### 4.7 The role of TRPA1

What do the peptidergic and non‐peptidergic C and Aδ neurons have in common that ablation of both can almost abolish the second phase? It is the expression of the nearly universal chemoreceptor‐channel TRPA1 that occurs in both subpopulations, even including LTM C‐units (Patil et al., 2018). Indeed, our single‐fiber recordings *ex vivo* provide a most similar picture as the ablation experiments *in vivo*: biphasic discharge activity in TRPA1 wildtype littermates upon formaldehyde 30 mM and only remnants of an early and short second phase left in knockout mice (Figure 8). The first phase, however, was less reduced, like in the ablation studies, indicating unspecific excitatory effects of formaldehyde which the small fibers share with the myelinated Aβ‐fibers that do not express TRPA1. Such minor unspecific effects also occur in the second phase of the *in vivo* formalin test on TRPA1 knockouts, even when only 0.5% (62 mM) solution is used which favors TRPA1‐dependent excitation (McNamara et al., 2007). In our hands, thick cutaneous Aβ‐fibers not expressing TRPA1 get excited from 30 mM on and only show one short phase of discharge before losing excitability (Figure 2). This indicates that in the usual (1–10%) formalin test the first phase will for the most part be due to unspecific excitatory effects in and around the injection bleb, while the second phase results largely from TRPA1 activation. The TRPA1‐independent release of CGRP occurred when the local concentration exceeded 10 mM formaldehyde in our skin and trachea preparations and also showed a sigmoidal concentration‐response that in the skin reached the TRPA1‐dependent magnitude at the saturating concentration of 100 mM (Figure 7). In keratinocytes, the above mentioned SERCA block by formaldehyde may account for this high degree of TRPA1 independent action in the skin, as the resulting cytosolic calcium increase leads to ATP release which can activate apposed sensory nerve endings through P_2_X_4_ receptor‐channels, as recently reported (Fischer et al., 2015; Sadler et al., 2020).

### 4.8 TRPA1‐independent excitation

What else may exert these “unspecific” TRPA1‐independent effects of formaldehyde? Any amount of indiscriminate posttranslational protein modifications with functional consequences is conceivable by electrophilic formation of hydroxyl‐methyl adducts, ‘methylene bridges’, cross‐linking, and finally by rapid membrane vesiculation (“blebbing”) as reported from histology (Fox et al., 1985; Kiernan, 2000; Thavarajah et al., 2012). To our knowledge, the earliest approach towards a cellular or molecular mechanism was made in Bertil Hille's laboratory; they found that 40 mM formaldehyde slowed the voltage‐dependent inactivation of sodium currents in frog muscle cells (Nonner et al., 1980). Although this could increase excitability in neurons, the effect would quickly be surpassed by the hyperpolarizing and blocking action in DRGs that already sets in at 1 mM formaldehyde and grows steeply with concentration (Fischer et al., 2014). Apart from TRPA1‐overexpressing CHO cells (Macpherson et al., 2007), the lowest formaldehyde concentration reported biologically effective caused a relaxation of the pre‐contracted murine superior mesenteric artery with an EC_50_ of 52 µM; endothelial TRPA1 and the NO/cGMP pathway are part of the mechanism. The authors suggest a role in postprandial gastrointestinal hyperemia and point to the similarity between gaseous formaldehyde and the gasotransmitters NO, H_2_S, and CO (Jin et al., 2019). The endogenous enzymatic production of formaldehyde as an intermediate in the amino acid metabolism amounts to almost 100 g per day in humans, leading to plasma levels around 20 µM formaldehyde (Szarvas et al., 1986). That TRPA1 is by far not the only target of modification is emphasized by the finding that formalin injection to the mouse hindpaw induced a massive neuronal overexpression of activating transcription factor 3 (ATF3), a reliable marker of nerve injury, in large numbers of DRG neurons with unmyelinated as well as myelinated fibers and independent of TRP channel expression (Bráz & Basbaum, 2010).

### 4.9 Na_V_1.7, TRPV4, ANO1, SERCA

One novel target has recently been identified by the reducing effect on both phases of the formalin test of the selective blocker phlotoxin‐1 of Na_V_1.7, the voltage‐gated sodium channel that normally triggers the action potentials in unmyelinated nerve endings and fibers; also synthetic Na_V_1.7 blockers have been shown effective in the formalin test (Matson et al., 2015; Nicolas et al., 2019). A voltage‐sensing action potential trigger, such as Na_V_1.7, is required for the transient excitatory effect in the first phase but also during the second phase, as TRP channels can just depolarize but not shape propagating action potentials. This also applies to another new target, the TRPV4 receptor‐channel involved in osmosensing and inflammatory pain. In TRPV4 knockout animals, the formalin test phase 2 is reduced, less so phase 1 (Chen et al., 2014). However, cation unselective TRP channels, when activated, also increase the cytosolic calcium concentration, and this alone can lead to neuronal excitation, for example by activating the calcium‐gated chloride channel ANO1 which is expressed in sensory DRG neurons; its genetic deletion halves the behavioral scores in phase 2 but leaves phase 1 unchanged (Lee et al., 2014). TRPA1 and Na_V_1.7, cooperating in excitation, are expressed along the axons in skin and peripheral nerves, but whether TRPV4 or ANO1 are functionally available there is not yet known to our knowledge (Nicolas et al., 2019; Weller et al., 2011). Even intracellular calcium release from or block of storage in the endoplasmic reticulum could activate excitatory mechanisms such as through ANO1. The already mentioned SERCA inhibition by formaldehyde, complete at 10 mM, raises the cytosolic calcium in keratinocytes and DRGs (Fischer et al., 2015). However, unmyelinated nerve fibers do not possess endoplasmic reticulum, in contrast to their cell bodies; whether the closely SERCA‐related plasma membrane calcium‐ATPase (PMCA) is also blocked by formaldehyde, enhancing intracellular calcium, is not yet known.

### 4.10 Ca_V_3.2, HCN2

What is available along the axons and in peripheral nerves is the low voltage‐activated T‐type calcium channel Ca_V_3.2 in the majority of C‐fibers, peptidergic and non‐peptidergic, including the LTM hair follicle receptors already discussed. In Emmanuel Bourinet's laboratory evidence has been accumulated over years that Ca_V_3.2 is a key player in adjusting the electrical excitability of the neurons; it amplifies depolarizing generator potentials evoked by any sensory transduction process so that the action potential threshold is eventually reached. Deleting Ca_V_3.2 expression (in Na_V_1.8‐expressing nociceptors) reduces the second phase of the formalin test, much less the first phase (François et al., 2015). A very similar amplifier function is attributed to the TTX‐resistant sodium channel Na_V_1.9; its genetic deletion reduces the second but not first phase and the stimulated CGRP release from the skin (Hoffmann et al., 2017; Lolignier et al., 2011). Last not least, the lowest voltage threshold of the potential amplifiers is due to HCN2, the unspecific cation inward channel actually activated by hyperpolarization but active at membrane resting potential and supporting generator potentials; its genetic deletion (again in Na_V_1.8‐expressing nociceptors) or block by ZD7288 reduces phase 2 but not phase 1 of the formalin test (Emery et al., 2011).

### 4.11 K^+^ channels

All these pro‐excitatory, TRPA1‐independent or secondary mechanisms of formaldehyde's actions seem to be predominantly involved in the second phase of the formalin test, supporting TRPA1‐mediated excitation or substituting for TRPA1 partly in case of its deletion. In contrast, the counterparts of excitatory, depolarizing ion channels, the hyperpolarizing potassium channels seem to affect the formalin test only weakly and both behavioral phases of the test to a similar extent. Calcium‐activated K^+^ channels (BK, SK) are expressed in sensory neurons of the DRGs, but their block by charybdotoxin and apamin applied together with formalin showed no effect (Bahia et al., 2005; Li et al., 2007; Ortiz et al., 2003). Also the block of the voltage‐gated K^+^ channels by 4‐aminopyridine and tetraethylammonium was reported ineffective in the formalin test (Ortiz et al., 2003). A species difference, rat versus mouse, may explain why genetic deletion of the shaker‐related slowly inactivating K^+^ channel K_V_1.1 (KCNA1, α subunit) did show effect, enhancing both phases of the formalin test (Clark & Tempel, 1998). The pharmacological blocker experiments did not really cover the M‐channel (KCNQ2/3, K_V_7.2), relevant in sensory neurons, for example in tuning C‐fiber cold sensitivity (Vetter et al., 2013). The anticonvulsant drug retigabine and the uricosuric compound benzbromarone are both able to activate the hyperpolarizing M‐channel which results in a reduction of both formalin test phases (Hayashi et al., 2014; Zheng et al., 2015). However, puzzling is that the genetic deletion of TRESK, one of the archaic two‐pore potassium channels expressed in non‐peptidergic DRG neurons (MrgprD^+^, LTM‐C), did not lead to an augmentation but to a minor reduction of phase 1 and early phase 2; these animals are conspicuous, though, as they also show reduced responsiveness to the TRPA1 agonist mustard oil (Castellanos et al., 2020).

### 4.12 Toxicokinetics

A final drug interaction with the formalin test may deserve discussion, as it sheds additional light on the toxicokinetics that govern the second phase. Agonists of the µ‐opioid receptor, such as fentanyl 2 µM, completely block the depolarization (KCl)‐evoked CGRP release from the isolated rat sciatic nerve (Mambretti et al., 2016). The ultrashort‐acting opioid remifentanil administered i.v. immediately before formalin injection ‐ much like the short‐acting local anesthetic (Dallel et al., 1995) ‐ abolished phase 1 behaviors, delayed phase 2 but did not reduce its magnitude. In addition, immediate paw swelling, plasma extravasation and blood flow increase were prevented by the transient antinociceptive opioid effect but occurred delayed at the time of phase 2 and with normal magnitude, because the excitotoxin, formalin, was still on its way along excitable nerve fibers (Taylor & Basbaum, 2000; Taylor et al., 1997). These vascular responses (‘neurogenic inflammation’) are well known functions of stimulated substance P and CGRP co‐release, and they expand widely over time around the formalin injection bleb, as visualized by Evans Blue spread and extravasation as well as by laser‐Doppler‐scanning; the injection bleb itself remains white for hours and shows no blood flow, probably due to the vasotoxic effect of the high local formaldehyde concentration (Fischer et al., 2014; Jin et al., 2019). *In vivo*, formalin‐evoked ‘axon reflex’ mechanisms certainly contribute to the rapid expansion of plasma extravasation (by substance P) and even more to the much larger area of vasodilatation (by CGRP). In addition, however, ongoing lateral diffusion and excitotoxic action of formaldehyde have to be postulated, as nerve fibers facing 30 µM (< 1% of 2.5% formalin) or more are first hyperpolarized and then rapidly blocked, disabling neuropeptide release (Fischer et al., 2014; Taylor & Basbaum, 2000).

## 5 CONCLUSION

*In vitro*, formaldehyde “penetrates rapidly and fixes slowly” (Fox et al., 1985), thus, diffusion in the isolated skin out of the restricting application chamber (in our setting) is the only way to explain the ongoing second‐phase discharge activity for at least 40 min that depends largely, but not entirely, on TRPA1 as a primary transducer. Our results leave no room for any, in the true sense, ‘inflammatory’ mechanisms of excitation by formalin. The complexities of the excitotoxic mechanisms and toxicokinetics discussed appear necessary and sufficient to account for the biphasic time course of behavior in the formalin test on rodents. It appears no longer justified and misleading to claim effects on ‘inflammatory pain’ of peripherally administered or acting drugs that affect the formalin test. Any uncontrollable drug effect on local blood flow, endothelial permeability, cell adhesion, extracellular matrix etc., takes influence on the re‐distribution of injected formalin in the skin, on dilution and clearance of diffusing formaldehyde (Fischer et al., 2014). And any unknown drug effect on the multiplicity of formaldehyde's excitotoxic actions can feign an antinociceptive specificity which is in fact as unselective as, for example, the local anesthetic action. The conditions are entirely different with central nervous system‐restricted drug actions. Irrespective of the peripheral mechanisms, the simple and amply validated formalin test provides a prolonged, largely nociceptive, input to the central nervous system, much like an injury or an acute inflammation, and a centrally acting drug can reliably show its antinociceptive efficacy and potency, appropriate controls provided for general anesthetic actions and motor impairment. No other algogenic compound than formalin (and methylgloxal) provides such long lasting pain‐related behavior, whether bi‐ or monophasic does not really matter.

## CONFLICT OF INTEREST

There is no conflict of interest.

## AUTHOR CONTRIBUTIONS

TH, FK and BR performed the single‐fiber recordings and evaluations (Figures 8, 2, 3), TIK and SKS the CGRP release experiments (Figure 7), KK the compound action potential recordings (Figure 5), PR and LL the multifiber recordings (Figure 4), AB the patch‐clamp and MJMF the calcium imaging (Figure 6), LK the behavioral studies (Figure 3A), and she wrote an early draft of the manuscript (1988). GC came (to Heidelberg) with the idea to study formalin effects on primary afferent level and did the *in vivo* experiments (Figure 1) together with PWR who supervised the further studies and wrote the final manuscript. MJMF performed most of the statistical analyses, designed and unified the figures, and edited the manuscript. All co‐authors provided intellectual input and agreed on the final manuscript. FK’s work was incorporated in a doctoral dissertation that fulfilled the requirements for promotion to Dr.med. at the University of Heidelberg.
